# Sequence Variation in HSP40 Gene among 16 *Toxoplasma gondii* Isolates from Different Hosts and Geographical Locations

**DOI:** 10.1155/2015/209792

**Published:** 2015-11-03

**Authors:** Zhong-Yuan Li, Jing Lu, Dong-Hui Zhou, Jia Chen, Xing-Quan Zhu

**Affiliations:** ^1^State Key Laboratory of Veterinary Etiological Biology, Key Laboratory of Veterinary Parasitology of Gansu Province, Lanzhou Veterinary Research Institute, Chinese Academy of Agricultural Sciences, Lanzhou, Gansu 730046, China; ^2^College of Animal Science and Veterinary Medicine, Heilongjiang Bayi Agricultural University, Daqing, Heilongjiang 163319, China; ^3^Guangdong Dahuanong Animal Health Products Co., Ltd., Yunfu, Guangdong 527400, China

## Abstract

*Toxoplasma gondii* with worldwide distribution has received substantial medical and scientific attentions as it causes serious clinical and veterinary problems especially for pregnant women and immunocompromised patients. Heat shock protein 40 (HSP40) plays a variety of essential roles in the pathogenesis of this protozoan parasite. In order to detail the genetic diversity of HSP40 gene, 16 *T. gondii* strains from different hosts and geographical locations were used in this study. Our results showed that HSP40 sequence of the examined strains was between 6621 bp and 6644 bp in length, and their A+T content was from 48.54% to 48.80%. Furthermore, sequence analysis presented 195 nucleotide mutation positions (0.12%–1.14%) including 29 positions in CDS (0.02%–0.12%) compared with *T. gondii* ME49 strain (ToxoDB: TGME49_265310). Phylogenetic assay revealed that *T. gondii* strains representing three classical genotypes (Types I, II, and III) were completely separated into different clusters by maximum parsimony (MP) method, but Type II and ToxoDB#9 strains were grouped into the same cluster. These results suggested that HSP40 gene is not a suitable marker for *T. gondii* population genetic research, though three classical genotypes of *T. gondii* could be differentiated by restriction enzymes *Msc*I and *Ear*I existing in amplicon C.

## 1. Introduction

*Toxoplasma gondii* infects almost all the warm-blooded animals including about one-third population of humans [1, 2] and can cause serious clinical diseases, especially in pregnant women and immunocompromised individuals such as tumor sufferers and AIDS patients [3, 4].* T. gondii* can also cause abortion and congenital toxoplasmosis in livestock, leading to considerable economic losses [5, 6].

Heat shock proteins (HSPs) involved in antigen presentation and cross-presentation play important roles in activation of immune-related cells such as macrophages, lymphocytes, and DCs [7–9]. As the important member of HSPs, HSP40 associated with DNA replication, protein folding, assembling and degradation, translocation across membranes, signal transduction, and endocytosis participates in the pathogenesis of apicomplexan parasites such as* Plasmodium falciparum* [10, 11]. Recent studies emphasized that different clonal types of* T. gondii* strains with diverse geographical distribution can cause different toxoplasmosis in animals and humans [12, 13]. In order to unveil the details of* T. gondii* genetic diversity, sequence variation of the type II HSP40*Tg*SIS1 (ToxoDB: TGME49_265310, previously named TGME49_065310) [14] among* T. gondii* isolates from different hosts and geographical regions was examined in this study.

## 2. Materials and Methods

### 2.1. *T. gondii* Isolates and gDNA Preparation

Sixteen* T. gondii* isolates harvested from different hosts and geographical locations were used in the present study (Table 1). Genomic DNA was extracted as normal and stored at −20°C till used.

### 2.2. PCR Amplification and Sequencing

**Figure 1:**
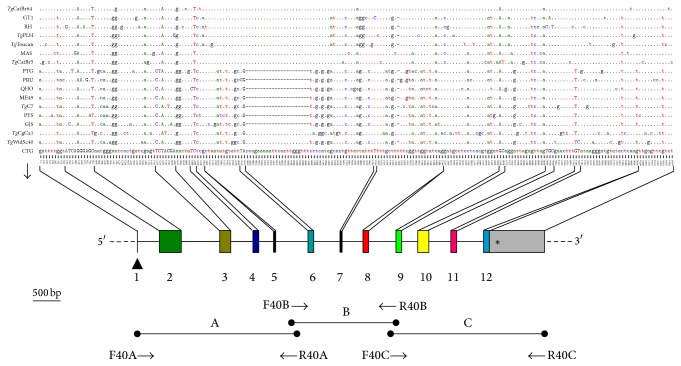
Sequence analysis of HSP40 gene among different* Toxoplasma gondii* strains. Color boxes indicate 12 extrons of HSP40 gene and the gray one stands for the region of 3′-UTR. Black triangle (▲) and asterisk (*∗*) indicate the translation start condon (ATG) and the stop one (TAG), respectively. Three pairs of specific primers (F40A/R40A, F40B/R40B, and F40C/R40C) used in this study were marked with arrows. Upper or lower case, respectively, indicates different nucleotide of extron or intron/3′-UTR. Point (.) and stub (–) among different nucleotides indicate identical or no nucleotide here compared with that of CTG strain (bottom line). The number beside the sequence stands for the variable sequence position for nucleotide, and black bar indicates 500 bp in length.

Three fragments (A, B, and C) (Figure 1) were separated based on the HSP40 sequence of* T. gondii* ME49 isolate (ToxoDB: TGME49_265310), and the amplifications were performed by PCR using three pairs of specific primers, respectively (Table 2). Thermal cycling conditions were according to the following protocol: initial denaturation at 94°C for 10 min followed by 35 cycles composing of 94°C for 1 min, 54.7°C (amplicon A), 66.8°C (amplicon B) or 64.0°C (amplicon C) for 45 s respectively, and 72°C for 2 min, and the additional extension step was carried out at 72°C for 10 min. Negative control without gDNA was included in each amplification. PCR amplifications were confirmed by agarose gel electrophoresis as previously described [15]. All the PCR products were purified using spin columns (Promega, Madison, USA), ligated with pMD18-T vector (TaKaRa, Dalian, China), and transformed into* E. coli* DH5*α* competent cells (Promega) according to the manufacturers' instructions. The positive colonies confirmed by PCR were sequenced by Shanghai Sangon Biological Engineering Biotechnology Company. All the experiments were run in triplicate.

### 2.3. Sequence Analysis and Phylogenetic Reconstruction

The sequences of all the examined* T. gondii* strains were amplified step by step and sequenced. Alignment analysis based on the obtained sequences including the reference one (ToxoDB: TGME49_265310) was carried out with Clustal X 1.83 [16], and the number of sequence variation compared with ME49 strain was calculated as previously described [17]. Phylogenetic reconstructions were performed by maximum parsimony (MP) method using PAUP^*∗*^ 4.0b10 [18], and 100 random addition searches using tree-bisection reconnection (TBR) were carried out for each MP assay. Bootstrap probability (BP) was calculated from 1,000 bootstrap replicates with 10 random additions per replicate in PAUP.

### 2.4. Characterization of* T. gondii* Isolates by PCR-RFLP

To evaluate whether HSP40 gene was suitable for genotyping of* T. gondii* isolates, PCR-RFLP method was also used in this study as previously described [19, 20]. All the PCR products of amplicon C were digested with two restriction enzymes* Msc*I and* Ear*I by incubating at 37°C for 4 h according to the manufacturer's instructions (NEB, Beijing, China). And the restriction fragments were separated by electrophoresis as previously described [21].

## 3. Results and Discussion

Our results showed that HSP40 gene of all the examined strains was between 6621 bp and 6644 bp in length spliced by amplicons A (2430 bp), B (2107–2130 bp), and C (2252 bp), and their A+T contents varied from 48.54% to 48.80% (Figure 1, Table 3). The alignment of all the 17 sequences revealed nucleotide mutations at 195 positions (0.12%–1.14%) in HSP40 genomic locations and 29 positions in CDS (0.02%–0.12%) in comparison with* T. gondii* ME49 strain (ToxoDB: TGME49_265310), which was lower than our previous reports of GRA5 [21], ROP38 [22], ROP47 [23], eIF4A [15], and other genes of* T. gondii*, such as GRA6 [24]. Moreover, 141 transitions (A↔G and C↔T) and 54 transversions (A↔C, A↔T, G↔T, and G↔C) (*R* = transition/transversion = 2.6) were also identified, and the distance of evolutionary divergence was 0.1%–1.0% among the examined* T. gondii* strains (Table 3).

**Figure 2:**
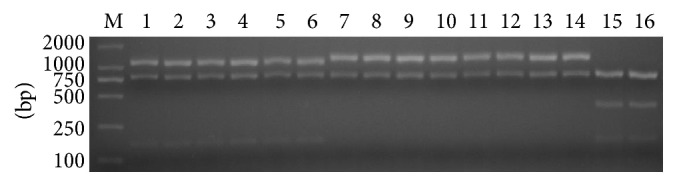
PCR-RFLP analysis based on the PCR products of amplicon C. Lane M indicates DL2000; lanes 1–16 stand for* T. gondii* Type I (GT1, RH, and* Tg*PLH);* Tg*Toucan, MAS, and* Tg*CatBr5, Type II (PTG, PRU, and QHO); ToxoDB#9 (*Tg*C7, PYS, and GJS),* Tg*CgCa1, and* Tg*WtdSc40, Type III (CTG); and* Tg*CatBr64, respectively.

Nucleotide polymorphisms analysis revealed two polymorphic restriction sites* Msc*I and* Ear*I in the sequence of amplicon C (2252 bp in length), which can differentiate three classical genotypes of* T. gondii* (Types I, II, and III) (Figure 2) [19, 20, 25, 26]. In brief, the PCR products of* Tg*Toucan, MAS,* Tg*CatBr5, and* T. gondii* Type I strains (GT1, RH, and* Tg*PLH) were digested into four segments (81, 165, 811, and 1195 bp); Type II strains (PTG, PRU, and QHO), ToxoDB#9 (*Tg*C7, PYS, and GJS),* Tg*CgCa1, and* Tg*WtdSc40 were composed of three parts (81, 811, and 1360 bp); and the PCR products of Type III (CTG) and* Tg*CatBr64 were cut into five sections (81, 165, 381, 811, and 814 bp). The results suggested that all the examined* T. gondii* strains could not be completely separated into their own groups by PCR-RFLP especially for ToxoDB#9 strains.

**Figure 3:**
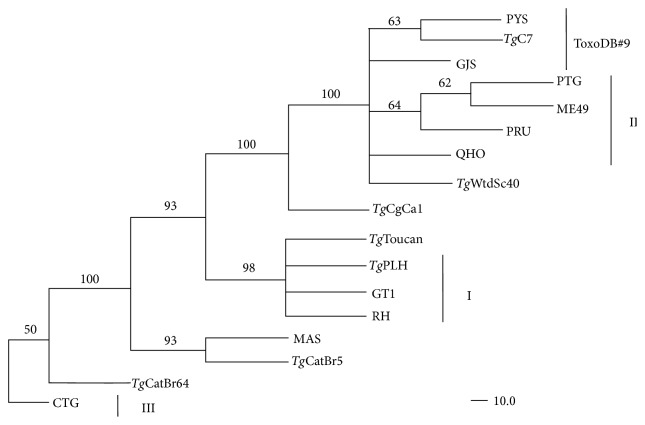
Phylogenetic analysis of HSP40 gene among 17* Toxoplasma gondii* strains using maximum parsimony (MP) method. Three clusters of the classical genotypes (Types I, II, and III) and ToxoDB#9 were denoted. The numbers along branches indicate bootstrap values (%).

Phylogenetic reconstruction was constructed based on HSP40 sequences of the 17* T. gondii* strains including* T. gondii* ME49 isolates (ToxoDB: TGME49_265310) (Figure 3) [18]. Our results showed that* T. gondii* strains belonging to Type II (PRU, QHO, ME49, and PTG), Type 12 (*Tg*WtdSc40), or ToxoDB#9 (PYS,* Tg*C7 and GJS) were grouped into the same cluster, whereas* Tg*Toucan (ToxoDB#52) was gathered into the cluster of Type I (RH, GT1, and* Tg*PLH), suggesting that the examined* T. gondii* strains could not be completely separated by MP method though three classical genotypes of* T. gondii* (Types I, II, and III) were clustered into different groups.

## 4. Conclusion

Our data suggested that HSP40 gene is not a suitable marker for* T. gondii* population genetic study, though three classical genotypes of* T. gondii* (Types I, II, and III) could be differentiated by polymorphic restriction endonuclease sites* Msc*I and* Ear*I existing in amplicon C.

## Figures and Tables

**Table 1:** Details of *Toxoplasma gondii* isolates used in this study.

Number	Isolate	Host	Geographical location	Genotype^*∗*^
1	CTG	Cat	United States	Reference, Type III, ToxoDB#2
2	*Tg*CatBr64	Cat	Brazil	Reference, ToxoDB#111
3	GT1	Goat	United States	Reference, Type I, ToxoDB#10
4	RH	Human	France	Reference, Type I, ToxoDB#10
5	*Tg*PLH	Pig	Henan, China	Type I, ToxoDB#10
6	*Tg*Toucan	Toucan	Costa Rica	Reference, ToxoDB#52
7	MAS	Human	France	Reference, ToxoDB#17
8	*Tg*CatBr5	Cat	Brazil	Reference, ToxoDB#19
9	PTG	Sheep	United States	Reference, Type II, ToxoDB#1
10	PRU	Human	France	Type II, ToxoDB#1
11	QHO	Sheep	Qinghai, China	Type II, ToxoDB#1
12	*Tg*C7	Cat	Guangzhou, China	ToxoDB#9
13	PYS	Pig	Panyu, China	ToxoDB#9
14	GJS	Pig	Jingyuan, Gansu, China	ToxoDB#9
15	*Tg*CgCa1	Cougar	Canada	Reference, ToxoDB#66
16	*Tg*WtdSc40	Deer	USA	Type 12, ToxoDB#5

^*∗*^Based on the results of Zhou et al. [25, 26] and Su et al. [20].

**Table 2:** Details of primers used in this study.

Primer	Sequence (5′-3′)
F40A	ATGGGGAAGGTAATCACATT
R40A	CGACTGGAACTATCGATTTC
F40B	TGTGGGGCGAGAGCCAGAGG
R40B	TGCAGAGGTGCCTTGCGTTT
F40C	CCCTGCATCGCGTGAGCTTC
R40C	AACGAGTGGAAAGCCCCCGT

Amplicons A, B, and C were amplified from different *T*. *gondii* strains by PCR using F40A/R40A, F40B/R40B, and F40C/R40C, respectively.

**Table 3:** Sequence characteristics of *Toxoplasma gondii* HSP40 including its expressed regions and introns.

Item	DNA	cDNA	CDS	Number of intron										
				1	2	3	4	5	6	7	8	9	10	11
Length (bp)	6621–6644	1620	1011	471	595	320	278	501	629–651	533-534	547	287	367	473
A+T (%)	48.54–48.80	45.12–45.56	45.20–45.70	44.80–45.65	48.40–48.91	45.62–46.56	45.68–46.40	51.70–52.10	53.90–54.99	48.78–50.28	51.74–52.47	54.70–55.75	45.78–46.32	47.36–47.78
Transition	141	36	26	7	15	5	3	8	14	12	10	9	6	16
Transversion	54	5	3	2	6	0	0	4	8	10	9	5	1	4
*R* ^*∗*^	2.6	7.2	8.6	3.5	2.5	—	—	2	1.7	1.2	1.1	1.8	6	4
Distance (%)	0.1–1.0	0.1–0.7	0–1.1	0–1.1	0–1.4	0–0.9	0–1.1	0–1.8	0–1.9	0–2.1	0–1.8	0–2.1	0–1.1	0.2–1.5

^*∗*^ *R* = transition/transversion; — means no data here.
